# Editorial: The roles of ncRNAs in the genome evolution and cross-kingdom regulation

**DOI:** 10.3389/fpls.2023.1133096

**Published:** 2023-02-03

**Authors:** Enhui Shen, Jun Zou, Jiandong Bao, Qian-Hao Zhu

**Affiliations:** ^1^ College of Agriculture and Biotechnology, Institute of Crop Science, Zhejiang University, Hangzhou, China; ^2^ Center for Bioinformatics and Big Data Technology, The Rural Development Academy, Zhejiang University, Hangzhou, China; ^3^ National Key Laboratory of Crop Genetic Improvement, Huazhong Agricultural University, Wuhan, China; ^4^ State Key Laboratory for Managing Biotic and Chemical Treats to the and Safety of Agro-Products, Zhejiang Academy of Agricultural Sciences, Hangzhou, China; ^5^ Black Mountain Laboratories, Commonwealth Scientific and Industrial Research Organisation (CSIRO) Agriculture and Food, Canberra, ACT, Australia

**Keywords:** ncRNAs, stress response, bioinformatic tools, gene interaction, genome evolution

Each plant genome transcribes a massive number of non-coding RNAs (ncRNAs), with some being constitutively transcribed and some being induced by abiotic and/or biotic stresses. ncRNAs are classified based on variable criteria, for example, house-keeping and regulatory ncRNAs, linear and circular ncRNAs. According to their sequence length, ncRNAs can be classified into three groups: small RNA (sRNA) with a length < 50 nucleotides (nt), long ncRNA (lncRNA) with a length > 200 nt, and intermediate-size ncRNA with a length between 50 and 200 nt (Wang et al., 2014). ncRNAs play vital roles in many aspects of the life cycle of plants, such as development, stress responses, interaction with other organisms and genome evolution (Sunkar et al., 2012; Li et al., 2017; Shen et al., 2020; Song et al., 2021). Since the discovery of the regulatory ncRNAs and with the advancing of sequencing technologies, extensive efforts have been devoted to identify ncRNAs, to investigate the scope of ncRNAs, and to understand the functionality of ncRNAs and their evolutionary dynamics at different level of plant lineages. This special issue aims to present the progresses on plant ncRNAs related to these topics and beyond. The articles compiled in this special issue cover the identification and characterization of ncRNAs responding to biotic stress and application of different forms of nitrogen, and of those associated with inter-subspecific heterosis. These studies included diverse plant species, from woody plant (poplar, *Populus x canescens*) to staple food crop (rice, *Oryza sativa*). Interaction among different types of ncRNAs as well as between ncRNAs and protein coding genes was interrogated to explore the potential regulatory roles of ncRNAs. More importantly, more than 100 tobacco (*Nicotiana tabacum*) lncRNAs were shown to contain short open reading frames (ORFs) encoding small peptides, with some being experimentally confirmed. The current available ncRNA databases and bioinformatic tools were summarized in one of the articles, providing a “one-stop shop” for the researchers who are interested in studies of plant ncRNAs.

Development and growth of plants depends on uptake of a diverse of elemental nutrients from soil, with nitrogen being one of the most critical nutrients influencing the growth and productivity of plants (Oldroyd and Leyser, 2020). Studying the roles of lncRNAs in response to application of different forms of nitrogen fertilizer will extend our understanding on the regulatory network associated with utilization of soil nitrogen. Based on strand-specific RNA-sequencing of RNAs isolated from roots of young plants of poplar *Populus x canescens* treated with three different nitrogen source, i.e., NH_4_NO_3_ (control), NaNO_3_, and NH_4_Cl, Zhou et al. performed systematic identification of lncRNAs, and identified differentially expressed (DE) lncRNAs between NaNO_3_ and NH_4_NO_3_ as well as between NH_4_Cl and NH_4_NO_3_. Many DE lncRNAs were found to be microRNA (miRNA) precursors and endogenous target mimics (eTMs) of miRNAs. They further constructed the gene regulatory networks involving lncRNA, mRNA, miRNA and eTM. Two regulatory modules involving miR171i or miR169b were confirmed based on RT–qPCR analysis of the transcript level of the miRNA targets (protein coding genes) that were transiently co-expressed with other components of the individual modules.

Heterosis is a fundamental biological phenomenon characterized by the superior performance of a hybrid compared with its parents, and has been widely applied in crop production for decades, particularly in rice and maize. miR2118-mediated cleavage of rice lncRNA *photoperiod sensitive male sterility 1* (*PMS1T*) generates phased small RNAs (phasiRNAs), which were found to be associated with photoperiod sensitive male sterility, a valuable trait in breeding two-line hybrid rice (Fan et al., 2016). But the molecular mechanisms behind the phenomenon of heterosis remain largely unknown (Shen et al., 2017). Wang and Wang conducted a genome-wide study on ncRNAs, including lncRNA, miRNA and circular RNA (circRNA), in inter-subspecific hybrid rice. On the basis of identification and characterization of the three types of ncRNAs in the hybrid and its parents, a total of 784 ncRNAs, including 169 miRNAs, 573 lncRNAs and 42 circRNAs, were found to be differentially expressed in the hybrid. The target genes of the DE miRNAs and lncRNAs and the parental genes of the DE circRNAs were functionally enriched in stress tolerance, plant growth and development. Based on comprehensive analyses of gene interactions, several ncRNA regulatory modules were proposed to play a potential role in rice inter-subspecific heterosis, although the speculations are yet to be confirmed experimentally.

By definition, ncRNAs are transcripts without protein coding potential, however, small ORFs are frequently predicted in many ncRNAs and several of such small ORFs have been demonstrated to encode functional peptides, i.e., small ORF-encoded peptides or SEPs, in plants (Lauressergues et al., 2015). Jin et al. performed a global identification of SEPs embedded in the tobacco (*N. tabacum*) lncRNAs responsive to *Spodoptera litura* (tobacco cutworm) infestation. Of the 302 SEPs identified to be derived from 115 lncRNAs based on mass spectrometry analysis, 61 responded to *S. litura* infestation, implying a potential role of these SEPs and/or the lncRNAs producing these SEPs in the interaction between *S. litura* and tobacco plants. Several of those SEPs were further characterized by prediction of 3D structure, investigation of subcellular localization based on GFP marker, and the expression of peptide based on western blotting. The results reported in this study suggest that SEPs encoded by lncRNAs could be a widespread phenomenon.

Given the importance of ncRNAs in plant biology, study on ncRNAs is now one of the hot Research Topics. Accordingly, many ncRNA-specific databases and research tools, including machine learning based methodologies, have been developed. Xu et al. summarized the progress achieved in the past decades on the development of ncRNA research tools, including ncRNA databases, bioinformatic tools for ncRNA prediction and characterization, and application of deep learning methods in ncRNA study. They also proposed the experimental criteria and methods to be used in verification of the identified ncRNAs and in investigation of ncRNA functionality. The article provides handy information necessary for investigation of plant ncRNAs. The information should be very useful for the plant ncRNA community, particularly for those who are new to the area. Moreover, the article also advocated for developing and designing user-friendly software and interfaces that can be easily adopted by biologists without sophisticated bioinformatics skills to facilitate studies on plant ncRNAs.

Overall, this special issue covers a range of active topics on studies of plant ncRNAs. In the future, to advance our understanding on the functionalities of plant ncRNAs in diverse biological processes, we need not only to identify novel ncRNAs but importantly to experimentally verify the predicted potential functions of the vast ncRNAs that have been identified, particularly the roles of lncRNAs and their interactions and evolutionary dynamics.

## Author contributions

All authors listed have made a substantial, direct, and intellectual contribution to the work and approved it for publication.
